# Targeted recombination of homologous chromosomes using CRISPR‐Cas9

**DOI:** 10.1002/2211-5463.13676

**Published:** 2023-07-26

**Authors:** Wonseok Son, Ki Wha Chung

**Affiliations:** ^1^ Department of Biological Sciences and BK21 Team for Field‐oriented BioCore Human Resources Development Kongju National University Gongju South Korea

**Keywords:** chromatin state, CRISPR targeting, *Drosophila* mutagenesis, *Lobe*, targeted recombination, transcriptional level

## Abstract

CRISPR mutagenesis is an efficient way to disrupt specific target genes in many model organisms. We previously devised a targeted CRISPR recombination method to generate intragenic recombinants of alleles in *Drosophila*. Here, we assessed the applicability of CRISPR targeting‐induced recombination to different genetic loci. We compared the ectopic recombination rates in the male germline by CRISPR targeting at two neighboring genetic loci within the genomic region that consists of the repressed chromatin domain of the *Lobe* gene, and the transcriptionally active domain of *PRAS40*. Targeting around the transcription initiation of *PRAS40* resulted in higher recombination rates of homologous chromosomes than targeting at the *Lobe* intron. Based on the efficient homologous recombination by CRISPR targeting observed around transcriptionally active loci, we further investigated targeted recombination between P‐elements that are inserted at different genomic locations. Male recombination by CRISPR targeting of P‐elements located proximally and distally to the *ebony* gene produced recombinants deficient for the intervening region of *ebony* transcription. Taken together, we suggest that targeted homologous recombination by CRISPR targeting may have specific genetic applications, such as generation of allelic combinations or chromosomal variations.

## Graphical abstract

Like the intragenic recombination of alleles shown in the figure, we addressed the targeted recombination of different homologous chromosomes using the CRISPR‐Cas9 targeting method as a useful genetic manipulation to produce specific allelic combinations or chromosomal variations. During evaluation of the ectopic recombination process, distinct recombination rates were identified between neighboring genetic loci of disparate chromatin states.

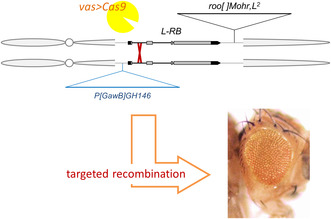

**Abbreviations**
Cas9: CRISPR associated protein 9
CRISPR: clustered regularly interspaced short palindromic repeats
DSB: double‐strand break
gRNA: guide RNA
HR: homology‐dependent repair
NHEJ: nonhomologous end‐joining
*e*
: *ebony*
*L*
: *Lobe*
*Mnat9*
: microtubule‐associated Nat9
*PRAS40*
: Proline‐rich Akt substrate 40 kDa

One of the advantages of *Drosophila* as a genetic model is easy performance of genome‐wide random mutagenesis and screening for an interested biological function with the help of useful genetic markers. Ionizing radiation was first applied in *Drosophila* to generate a variety of chromosomal variations, and random incorporation of modified transposons has been accumulated to access almost every gene in the fly genome. More recently, targeted mutagenesis of CRISPR‐Cas9 protocol has become widely applied for site‐directed disruption and modification of an interested gene [1, 2, 3, 4].

During identifying the *Lobe* (*L*) eye‐causing gene in the previous study, we designed a targeted recombination at specific genomic sites by using CRISPR targeting in the male germline to combine intragenic mutations in the *cis* position [5]. The idea of targeted recombination was based on the finding that CRISPR mutagenesis is accompanied by homology‐dependent repair (HR) of targeted DNA breaks and homologous recombination in the meiotic germline expressing Cas9.

The CRISPR‐Cas9 targeting is dependent on endogenous repair systems to respond to the ectopic double‐strand breaks (DSBs) generated by the Cas9 nuclease. The DSB repair responses consist mainly of two separate and competing repair pathways: HR and the nonhomologous end‐joining (NHEJ). Compared to HR, the NHEJ is the faster and more efficient DSB repair response, and most imprecise repair results during CRISPR mutagenesis have conventionally been regarded as InDel mutations derived through the NHEJ repair pathway in *in vitro* cultured cells [6]. Mutagenesis results by CRISPR targeting in *Drosophila* have also been attributed largely to the error‐prone NHEJ repair [7]. However, we confirmed that germline repair of induced DSBs during CRISPR mutagenesis follows the HR pathway predominantly over NHEJ by using deficiency chromosomes that cover or uncover the targeted region [5]. Based on the HR‐dependency of CRISPR targeting events in the germline, we could induce intragenic recombination of alleles of different homologous chromosomes in the protocol of targeted recombination.

In the present study, we further tested the applicability of the targeted recombination beyond the *L* region and compared the ectopic recombination rates in genetic loci that may feature different chromatin accessibility in the male germline. Different from achiasmic males [8], crossover distribution during *Drosophila* female meiosis is influenced by meiotic chromatin states and associated DNA motifs to determine recombination rates [9, 10, 11]. Several mutageneses have included ectopic male recombination, such as the P‐element transposition‐induced recombination as an application of gene mapping [12]. However, there is little known association between chromatin state and mutagenesis rates in the male recombination. We addressed different recombination rates between CRISPR targeting at neighboring genetic loci of different transcriptional levels in this study. In addition, we also assessed the ectopic recombination by targeting at P‐element sequences that are positioned in different genomic locations. Consequently, we suggest the targeted recombination between homologous chromosomes as an efficient way of genome engineering for specific genetic purposes.

## Materials and methods

### *Drosophila* strains

*L* ^*2*^ dominant mutation was described by Son and Choi [5], and *mei‐218* ^*6*^ for ectopic meiotic NHEJ was described by Joyce *et al*. [13]. P‐element insertions of *P[GawB]GH146*, *P[EPgy2]EY10689*, *P[EP]G5381*, and *P[EPgy2]EY20305* were obtained from the Bloomington *Drosophila* Stock Center (BDSC, Bloomington, IN, USA; stock numbers are 30026, 20201, 30155, and 22382, respectively). The Cas9 sources [14] were obtained from BDSC (51323, 54590).

### Transgenic flies

Guide RNA (gRNA) sequences are listed in Table 1, and the targeting sites in the *L* and *PRAS40* region are shown by open triangles in Fig. 2A. gRNA constructs were cloned in *pBFv‐U6.2* or *pCFD4‐U6:1_U6:3*, then transgenic strains were generated following the strategy described by Port *et al*. [4, 14].

**Table 1:** Guide RNA sequences used in the study. ^1,2,3,4,5^gRNA target positions are represented by open triangles with numbers in Fig. 2A.

Transgenic strains	Target sequences	Targeting loci
*P[U6‐Mnat9]*	5′‐gtcgctccagggtgacctcg‐tgg‐3′ 5′‐gaccctgtgacccagtattt‐tgg‐3′	*Mnat9*
*P[U6‐RBit1]* ^1^ *P[U6‐RBit2]* ^2^	5′‐gtctaagttccactctaatt‐ggg‐3′ 5′‐ggtcaacaccaaatgttgcc‐cgg‐3′	*L*
*P[U6‐P405u]* ^3^ *P[U6‐P40ita]* ^4^ *P[U6‐P40itb]* ^5^	5′‐aattaccgttatcagctaac‐cgg‐3′ 5′‐atggaagggcaagaagaaat‐agg‐3′ 5′‐cagtgggtggacgtggtctc‐ggg‐3′	*PRAS40*
*P[U6‐P53]* *P[U6‐P31]* *P[U6‐P32]*	5′‐gtgcacgtttgcttgttgag‐agg‐3′ 5′‐aagtggatgtctcttgccga‐cgg‐3′ 5′‐ttaacccttagcatgtcccgt‐ggg‐3′	P‐element

### Male recombination between homologous chromosomes by CRISPR targeting

**Fig. 1:**
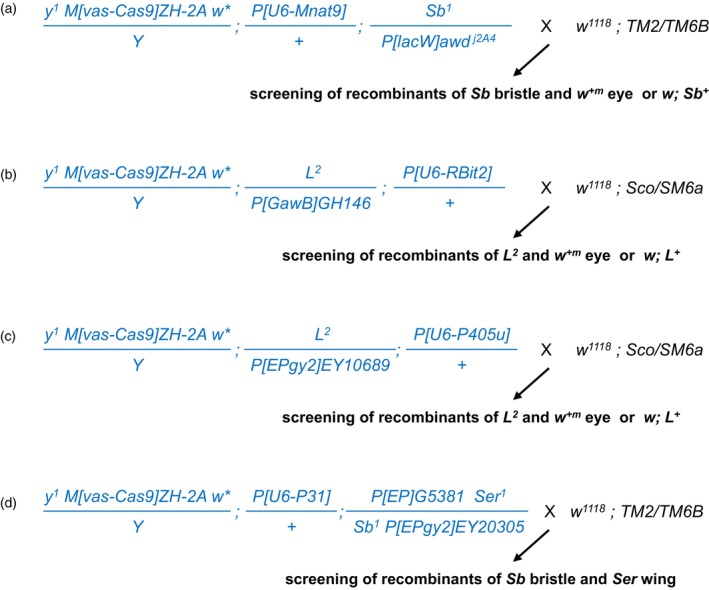
Genetic schemes for F2 screening of the targeted recombination. All the mutagenic F1 males are presented in blue. The mutagenic males were crossed with balancer stocks for the next recombinant screening in the F2 progeny. (A–C) Targeting at genetic loci of *Mnat9*, *L*, or *PRAS40*. Recombinants showing both or none of the dominant genetic markers of paternal homologous chromosomes were counted among the F2 progeny. (D) Targeted recombination between two different P‐elements located proximally or distally to the *e* transcription.

Prior to the targeted recombination crosses, each homologous chromosome to be recombined was prepared in double mutant stocks carrying the germline source of Cas9 (*M[vas‐Cas9]ZH‐2A*, *vas‐Cas9* in short) or gRNA transgenes. The G0 double mutant flies were crossed to collect the mutagenic F1 males that are transheterozygous for genetic markers or P‐element insertions and that are bound to express *Cas9* and specific gRNA transgenes in the germline. The mutagenic F1 animals were crossed with the second chromosomal or the third chromosomal balancer stocks for next screening of recombinants among the F2 progeny (Fig. 1).

## Results and discussion

### Variation in the targeted recombination rate by genomic loci

We previously devised a targeted recombination between *L* ^*2*^ mutation and *L‐GAL4* insertions to test whether intrinsic reporter activities of the GAL4 insertions can be ectopically induced by *L* ^*2*^‐specific *roo[]Mohr* transposon in *cis* position [5]. Since two mutation sites are too near to expect spontaneous recombinants of the *L* alleles by conventional meiotic recombination, we designed a CRISPR mutagenesis to induce targeted recombination within the short genomic distance, based on the finding that the HR‐dependent CRISPR targeting in the germline can trigger male recombination around target sites. Thereafter, we further evaluated whether the ectopic male recombination is applicable in other genetic loci beyond the *L* region, especially by comparing recombination rates by different genomic loci.

During the screening of the *L* ^*2*^‐reporters, we could collect recombinants only in the progeny of about 11.9% crosses (8 of 67 fertile crosses) of mutagenic F1 males that were transheterozygous for two *L* alleles, carrying both transgenes of *Cas9* and specific gRNA expressions. Finally, only eight independent recombinant lines were established after screening ~4000 F2 animals. Despite successful isolation of the expected recombinants of *L* alleles, the result of low recombination rates questioned the mutagenesis efficiency of the CRISPR targeting‐induced recombination in other applications besides the *L* region.

Regarding the low recombination output, we noticed that the *L* region is highly enriched with chromatin domains for the Pc‐mediated repression revealed by cultured cell‐based analyses [9, 15, 16]. For the natural meiotic recombination in the female germline, genome‐wide analyses of genomic features have evidenced that the recombination rate variation is dependent on the chromatin accessibility for DSB formation [9, 10]. Similarly, our results for targeted recombination in the male germline might have been affected by the chromatin state of target sites for DSB breaks. Although the available chromatin information of *L* region was based on analyses in cultured cell lines, we reasoned that the genomic region is transcriptionally inactive also in germ cells because we could not identify any detectable reporter activity of *L* in the reproductive organ.

Thereafter, we assessed the targeted recombination protocol in another genomic region of which the chromatin domain consists of active transcription motifs (at *Mnat9*) [17]. The chromatin states deduced from histone modifications and associated genomic motifs in the *Mnat9* region indicate a high level of chromatin accessibility as well as the elevated transcriptional level of the corresponding gene [9, 16, 18]. In addition to the cultured cell‐based genomic features, high‐throughput expression databases such as FlyAtlas2 and modENCODE indicate the highest level of *Mnat9* transcription in the male testis. Therefore, we tested the ectopic male recombination at the *Mnat9* locus that probably sustains the accessible chromatin state in the male germline.

The CRISPR targeting result at the *Mnat9* region was in sharp contrast with that of the *L* region. Virtually all F1 mutagenic males targeting at *Mnat9* sequences gave rise to at least a recombinant of both genetic markers from paternal homologous chromosomes (Fig. 1A). We could frequently discover those recombinants in the F2 progeny. The final percentage of recombinants among the F2 animals was highly estimated at 6.5% (240 recombinants among a total of 3695 F2 adults).

### Different recombination rates between CRISPR targeting at different chromatin domain regions

As described above, we confirmed a significant difference in recombination rates between CRISPR targeting at *L* and *Mnat9* loci, which genomic regions are distinguished by disparate chromatin states and transcriptional levels in cultured cells and probably in the male germ cells also. Those recombination rates were estimated from CRISPR mutagenesis in the male germline expressing Cas9, which prompted HR repair of the targeted DNA breaks and homologous recombination [5]. However, those loci are in different chromosomes, and in different relative chromosomal positions to centromeres that may affect recombination rates over the target sites.

**Fig. 2:**
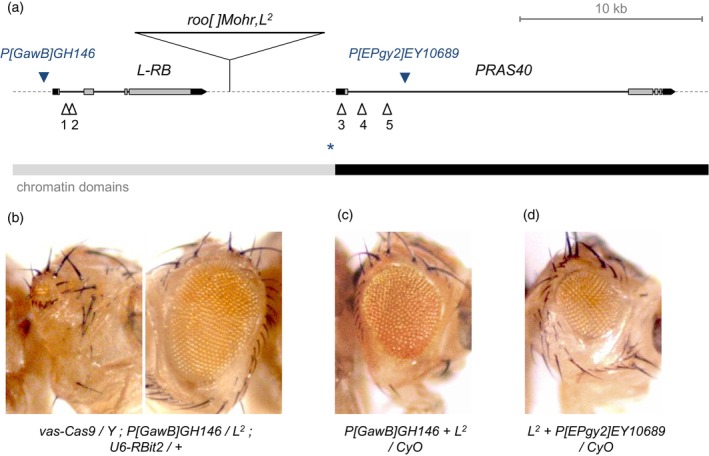
Comparison of targeted recombination rates between two adjacent genes of disparate chromatin states. (A) Genomic map around *L* and *PRAS40* genes. Only a short transcript is represented for *L* transcription in the map (*L‐RB*). The *L* ^*2*^‐specific mutant form of *roo[]Mohr* transposon is presented in the intergenic region between *L* and *PRAS40* [5]. Blue triangles indicate P‐element insertions in upstream sequences of *L‐RB* (*P[GawB]GH146*) and in the first intron of *PRAS40* (*P[EPgy2]EY10689*). Open triangles are the gRNA target sites that were used for targeted recombination between P‐element insertions and the *L* ^*2*^ eye phenotype in this study. Site numbers correspond to target sequences and transgenic strains presented in Table 1. A possible insulator element [24] is indicated as a blue asterisk in the upstream sequences of *PRAS40*. Chromatin domains are quoted from the FlyBase [9, 16]: the gray bar represents transcriptionally silent and a Polycomb‐mediated repressed region, and the black bar represents transcriptionally active euchromatin of the active promoter or actively transcribed region. (B) A mutagenic F1 male of targeting at the *L* region. The Cas9 source under the *vasa* promoter used in this study for the germline expression (*M[vas‐Cas9]ZH‐2A*) [14] is also active in developing eyes, thus generated frequent phenotypic mosaicism for the *L* ^*2*^ mutation located distally to CRISPR target sites. Both the left and right eyes of an individual are presented to show the phenotypic mosaicism. (C,D) Recombinants of both dominant markers (*L* ^*2*^ eye and *w* ^*+m*^ eye color) were collected among the F2 progeny of targeted recombination between the *L* ^*2*^ mutation and P‐element insertions in *L* (C) and *PRAS40* (D).

To compare the targeted recombination between genomic loci of different chromatin states but with little genetic influence by different regions, we utilized the *L* region that is just flanked by a transcriptionally active gene, *PRAS40* (Fig. 2). In the region, the *L* gene and its neighboring *PRAS40* are only about 7 kb apart between those open reading frames. However, genomic analyses have identified disparate chromatin states between those neighboring genes, as revealed by genomic data from cultured embryonic or larval cell lines [9, 16] (Fig. 2A). Consistent with the genomic features, *L* transcription becomes greatly reduced after embryogenesis, but a high transcription level of *PRAS40* sustains in a majority of proliferating cells [5, 19]. A CTCF binding site within the intergenic sequences between *L* and *PRAS40* may separate the distinct chromatin states between two genes (Fig. 2A). In addition to the cultured cell‐based features, RT‐PCR analysis verified significant *PRAS40* expression also in adult testis [19]. Hence, we reasoned that those transcriptional and chromatin features are probably shared between high‐proliferating and less‐differentiated cultured cells and spermatogonia in the male germline. Utilizing the genomic organization that disparate chromatin states are neighboring, we compared CRISPR targeting at those adjacent target sites in the *L‐PRAS40* region where different chromatin accessibility is expected.

gRNA target sites were chosen in the first *L* intron and around the first exon of *PRAS40* (open triangles in Fig. 2A). Recombination rates were assayed by using P‐element insertions on two genes (with a genetic marker of *w^+m^* eye color expressed by those P‐elements) and the *L* ^*2*^ eye phenotype, which is caused by mutations in the 9 kb‐long *roo[]Mohr* transposon that is located in the intergenic region between *L* and *PRAS40*. We collected the mutagenic F1 males that are transheterozygous for the *L* ^*2*^ mutation and P‐element insertions (*P[GawB]GH146* and *P[EPgy2]EY10689* that are on *L* and *PRAS40*, respectively) carrying a germline source of Cas9 and each specific gRNA transgene (Figs 1B,C, and 2B). Then the F2 progeny was screened for the recombinant eye phenotype (Fig. 2C,D).

Similarly to natural meiotic recombination rates dependent on chromatin accessibility in the female germline, we identified a significant difference in recombination rates between CRISPR targeting at the adjacent genomic loci of *L* and *PRAS40* (Table 2). While collecting recombinants from less than 10% of mutagenic F1 males targeting at the *L* intron, we could find at least a targeted recombinant in the F2 progeny from more than half of mutagenic F1 males where CRISPR targeting was around the first *PRAS40* exon. Counted numbers of F2 recombinants showed low recombination rates by CRISPR targeting at the repressed chromatin domain of the *L* region, but much higher efficacy of targeted recombination at the neighboring transcriptionally active region of *PRAS40* (Table 2).

**Table 2:** Different recombination rates between CRISPR targeting at the adjacent genomic loci of *L* and *PRAS40*. gRNA target sites are represented in Fig. 2A. The mutagenic F1 males were individually crossed with five to 10 females of the second chromosomal balancer stock, and were examined by the ratio of mutagenic crosses showing a recombinant at least in the F2 progeny.

Genotype of the mutagenic F1 male	Percentage of the mutagenic F1 males that produced a recombinant F2 at least	Ratio of phenotypic recombinants in the F2 progeny^a^	gRNA target sites
*vas‐Cas9/Y; L* ^*2*^ */P[GawB]GH146; P[U6‐RBit1]/+*	5.1% (5/98)	1.7% (17/991)	The first intron of *L‐RB*
*vas‐Cas9/Y; L* ^*2*^ */P[GawB]GH146; P[U6‐RBit2]/+*	8.6% (7/81)	2.6% (21/801)	The first intron of *L‐RB*
*vas‐Cas9/Y; L* ^*2*^ */P[EPgy2]EY10689; P[U6‐P405u]/+*	97.6% (40/41)	9.4% (157/1676)	5′ UTR of *PRAS40*
*vas‐Cas9/Y; L* ^*2*^ */P[EPgy2]EY10689; P[U6‐P40ita]/+*	61.2% (30/49)	7.0% (101/1437)	The first intron of *PRAS40*
*vas‐Cas9/Y; L* ^*2*^ */P[EPgy2]EY10689; P[U6‐P40itb]/+*	72% (36/50)	6.8% (111/1621)	The first intron of *PRAS40*

^a^ The recombinant ratio was calculated only in the F2 progeny of selected F1 mutagenic animals that produced a phenotypic recombinant at least.

Because genetic markers for the recombination assay were close enough to ignore possibility of spontaneous meiotic recombination in the female germline, we also collected F1 females of which germline was mutagenic as expressing *Cas9* and specific gRNAs and counted F2 recombinants by the CRISPR targeting. From the mutagenic F1 females, we could also collect targeted recombinants with similar or lower recombination rates (Table 3).

**Table 3:** Recombination rates by CRISPR targeting in the male or female germline, or with *mei‐218* ^*6*^ mutation in the male germline. The mutagenic F1 males or females were individually crossed with the second chromosomal balancer stock. Recombination rates are represented by the ratio of mutagenic crosses that produced recombinant F2 animals.

Genotype of the mutagenic F1 animal	Percentage of the mutagenic F1 animals that produced a recombinant F2 at least	Description
*vas‐Cas9/Y; L* ^*2*^ */P[GawB]GH146; P[U6‐RBit2]/+*	10.4% (5/48)	Targeting at the first intron of *L‐RB*; in the male germline
vas‐Cas9/w*; L ^*2*^ */P[GawB]GH146; P[U6‐RBit2]/+*	3.9% (2/51)	Targeting at the first intron of *L‐RB*; in the female germline
*mei‐218* ^*6*^ *vas‐Cas9/Y; L* ^*2*^ */P[GawB]GH146; P[U6‐RBit2]/+*	4.7% (2/43)	Targeting at the first intron of *L‐RB*; *mei‐218* ^*6*^ mutation in the male germline
*vas‐Cas9/Y; L* ^*2*^ */P[EPgy2]EY10689; P[U6‐P405u]/+*	92.0% (46/50)	Targeting at the 5′UTR of *PRAS40*; in the male germline
vas‐Cas9/w*; L ^*2*^ */ P[EPgy2]EY10689; P[U6‐P405u]/+*	66.0% (33/50)	Targeting at the 5′UTR of *PRAS40*; in the female germline
*mei‐218* ^*6*^ *vas‐Cas9/Y; L* ^*2*^ */ P[EPgy2]EY10689; P[U6‐P405u]/+*	80.0% (44/55)	Targeting at the 5′UTR of *PRAS40*; *mei‐218* ^*6*^ mutation in the male germline
*vas‐Cas9/Y; L* ^*2*^ */P[EPgy2]EY10689; P[U6‐P40itb]/+*	59.6% (31/52)	Targeting at the first intron of *PRAS40*; in the male germline
vas‐Cas9/w*; L ^*2*^ */ P[EPgy2]EY10689; P[U6‐P40itb]/+*	59.2% (29/49)	Targeting at the first intron of *PRAS40*; in the female germline
*mei‐218* ^*6*^ *vas‐Cas9/Y; L* ^*2*^ */ P[EPgy2]EY10689; P[U6‐P40itb]/+*	48.0% (24/50)	Targeting at the first intron of *PRAS40*; *mei‐218* ^*6*^ mutation in the male germline

### The targeted recombination rate was not affected by a *mei‐218* mutation in the male germline

Our previous data suggested that germline repairs of induced DSBs during CRISPR mutagenesis follow the HR pathway predominantly over NHEJ. These mutagenesis results might be attributed to meiosis‐specific regulations of DSB repairs in the germline. The choice between competing DSB repair pathways is affected by multiple regulatory mechanisms depending on cell cycle or cellular contexts [20, 21, 22]. For example, during meiotic DSB repairs yielding homolog crossovers to preserve genetic adaptability of an organism, the homologous repair pathway is promoted while the alternative erroneous NHEJ is suppressed. The NHEJ‐inhibiting regulations have also been identified in *Drosophila* oogenesis, for an example of ectopic NHEJ phenotypes associated with a mutation of *mei‐218* encoding an MCM complex gene [13].

We tested the *mei‐218* mutation causing ectopic NHEJ and examined a modification in the targeted recombination rate by a probable alteration of DSB repair regulation in the male meiosis, which is achiasmic normally in *Drosophila* [8]. However, no significant difference in rates of the ectopic male recombination was detected when the mutagenic F1 males carried the *mei‐218* mutation (Table 3).

### Targeted recombination between different P‐element insertions

Based on the efficient recombination results at genomic loci of high chromatin accessibility, we assessed the targeted recombination between different genomic loci by targeting P‐element insertions. It has been well known that P‐element preferentially inserts the promoter region of a subset of genes, which is liable to high chromatin accessibility for interaction with various transcriptional factors [23]. We tested CRISPR targeting at P‐element sequences located at different genomic sites to induce an ectopic recombination of different genomic loci. A variety of modified P‐elements have been developed for each specific purpose, and most of the variant constructs share common sequences in the terminal part of the transposon. We choose gRNA target sites within the sequences that are likely conserved among diverse P‐element constructs.

**Fig. 3:**
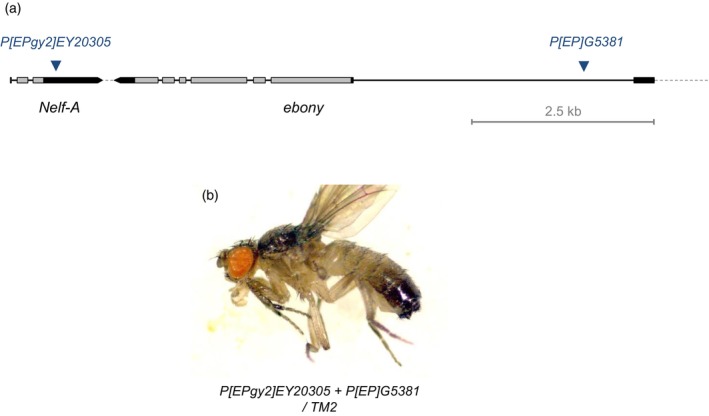
Targeted recombination between P‐element insertions at different genomic locations. (A) Genomic map of the *ebony* region and P‐element insertions. Blue triangles indicate P‐element insertions in the 5′ side (*P[EP]G5381*) and the 3′ side (*P[EPgy2]EY20305*) of the *ebony* gene. Targeted recombination of those two P‐elements is liable to generate a specific deletion mutant lacking the entire *ebony* transcription. (B) Consistently, the CRISPR targeting at those two P‐elements on both sides of *ebony* produced recombinants of the gene mutant body color.

We assessed the targeted recombination between P‐elements in the genomic region around *ebony* (*e*). We conducted the ectopic male recombination between two P‐element insertions, *P[EPgy2]EY20305* and *P[EP]G5381* that are located proximally and distally to the *e* gene, respectivlely. The genomic distance between those insertions is about 7.2 kb long, covering the entire *e* transcription (Fig. 3A). Those P‐element insertions were recombined beforehand with dominant genetic markers of *Sb* bristle or *Ser* wing to identify the P‐element recombination. We collected 52 mutagenetic males that were transheterozygous for those P‐element insertions, and crossed with a third chromosomal balancer for screening of F2 progeny (Fig. 1D). However, the majority of those mutagenic F1 males were sterile, unexpectedly, and we could collect few F2 progeny only from 22 mutagenic males.

Finally, we could collect two independent recombinants showing both genetic markers of paternal homologous chromosomes (Fig. 3B). The recombinants displayed the *e* mutant body color with third chromosomal balancers carrying an *e* mutation, as evidencing deletion of the *e* gene transcription by the P‐element recombination. PCR amplification and sequence analysis confirmed the distal end of *P[EP]G5381* and the proximal end of *P[EPgy2]EY20305*. But the genomic sequences proximal to *P[EP]G5381* and distal to *P[EPgy2]EY20305* were not amplified by PCR, consistent with the *e* phenotype of the recombinants.

The recombination rates of the targeting at P‐element sequences were much lower even than CRISPR targeting at the *L* region. The results were due in part to unexpected developmental phenotypes of the mutagenic F1 animals with the reduced F2 progeny. The developmental lesions might be due to off‐target effects of the gRNA target sequences at P‐element that we applied. Different gRNA targets at P‐element sequences should be evaluated to address more efficient P‐element recombination in next works.

Here, we presented several examples of the targeted recombination between homologous chromosomes in the male germline by an application of CRISPR‐Cas9 targeting. We could address that the ectopic male recombination is applicable at different genetic loci of *Mnat9* or *PRAS40* beyond the *L* region. During the recombination works, we noticed a genetic effect of different recombination rates by targeting loci. Based on the analysis of female meiotic recombination [10], we hypothesized that the difference might be due to the disparate chromatin accessibility for DSB formation of the targeting loci in the male germline. Consistently, a significant difference in the targeted recombination rates was identified in a genomic region of two adjacent genetic loci that show distinct levels of transcription in the male reproductive organ. And another ectopic recombination between different P‐element insertions was assessed in this study suggesting that the CRISPR targeting‐induced ectopic recombination is applicable to generate long‐distance mutations beyond a target site.

Taken together, the present results of targeted recombination we addressed the ectopic male recombination of different homologous chromosomes by using CRISPR targeting as a useful genetic manipulation to produce specific allelic combinations or chromosomal variations.

## Conflict of interest

The authors declare no conflicts of interest.

### Peer review

The peer review history for this article is available at https://www.webofscience.com/api/gateway/wos/peer‐review/10.1002/2211‐5463.13676.

## Author contributions

WS conceived the project, acquired the data, and drafted the article, KWC supervised the project and acquired funds.

## Data Availability

The data presented in this study are contained within the article. The data that support the findings of this study are available from the corresponding author upon reasonable request.
